# Super Resolution Infrared Thermal Imaging Using Pansharpening Algorithms: Quantitative Assessment and Application to UAV Thermal Imaging

**DOI:** 10.3390/s21041265

**Published:** 2021-02-10

**Authors:** Javier Raimundo, Serafin Lopez-Cuervo Medina, Juan F. Prieto, Julian Aguirre de Mata

**Affiliations:** Departamento de Ingeniería Topográfica y Cartográfica, Escuela Técnica Superior de Ingenieros en Topografía, Geodesia y Cartografía, Universidad Politécnica de Madrid, Campus Sur, A-3, Km 7, 28031 Madrid, Spain; s.lopezc@upm.es (S.L.-C.M.); juanf.prieto@upm.es (J.F.P.); julian.aguirre@upm.es (J.A.d.M.)

**Keywords:** thermal imaging, infrared, pansharpening, resolution enhancement, multispectral, super-resolution, remote sensing

## Abstract

The lack of high-resolution thermal images is a limiting factor in the fusion with other sensors with a higher resolution. Different families of algorithms have been designed in the field of remote sensors to fuse panchromatic images with multispectral images from satellite platforms, in a process known as pansharpening. Attempts have been made to transfer these pansharpening algorithms to thermal images in the case of satellite sensors. Our work analyses the potential of these algorithms when applied to thermal images from unmanned aerial vehicles (UAVs). We present a comparison, by means of a quantitative procedure, of these pansharpening methods in satellite images when they are applied to fuse high-resolution images with thermal images obtained from UAVs, in order to be able to choose the method that offers the best quantitative results. This analysis, which allows the objective selection of which method to use with this type of images, has not been done until now. This algorithm selection is used here to fuse images from thermal sensors on UAVs with other images from different sensors for the documentation of heritage, but it has applications in many other fields.

## 1. Introduction

The use of thermal cameras with a sensor that is sensitive to the long-wave thermal infrared part of the electromagnetic spectrum (9–14 micrometres) is becoming increasingly widespread. However, unlike other kinds of sensors such as visible spectrum range RGB cameras, the resolution of even the most advanced commercial sensors, that are sensitive to wavelengths usually between 2.5 and 15 µm, does not exceed the megapixel frontier. This is due to technical limitations: the miniaturization of the microbolometers, the elements that react to incoming infrared thermal waves, is inversely proportional to the signal-noise ratio [1]. It can reasonably be assumed that the resolution of thermal sensors will not equal that of other sensors (visible and near-infrared spectrum range) in the short and medium term [2].

Our work studies the quality of the results when we set out to increase the resolution of thermal images by fusing them with images from another sensor. This is particularly interesting as it is quite common to take thermal imaging simultaneously with other visible spectrum sensors. It is essential to visually inspect the study zone at the time the thermal data is taken, as objects in thermal imaging lack contrast, making it difficult to identify the focus. That is the reason almost every thermal sensor is combined with visible spectrum cameras to assure the right frame of capture.

Since the 1970s a variety of algorithms have been developed in remote sensing to improve the resolution of one type of low-resolution sensors with information from images with a higher resolution. These procedures are called pansharpening. This name was selected as these algorithms originally improved the low resolution of multispectral images using the panchromatic images taken by both satellite-mounted sensors [3].

Although pansharpening procedures are widely known, the first approaches to merging thermal and RGB images to enhance the resolution of the original thermal image involved applying the intensity-hue-saturation (IHS) pansharpening algorithm [4,5]. Other authors subsequently conducted research combining information from high-resolution visible spectrum images with thermal images obtained from terrestrial sensors [6,7,8].

The industry’s strategies to enhance thermal imaging include the development by the thermal camera maker FLIR of the Ultramax© technology, which combines numerous shots (16 shots per second), each slightly different from the other due to the inevitable movements and vibrations during the capture process. This proposed solution achieves a twofold improvement in the resolution [9].

Another manufacturer, InfraTec, devised a hardware solution with a fast-rotating wheel, which allows four images to be taken in rotation, which are fused in the final image [9].

Other approaches include Deep Learning techniques applied to this problem, introducing RGB images as part of the established network architecture [10]. The limitation of these approaches is that they require a prior training phase, and the extrapolation of this training may not be adequate in all situations.

In the field of enhancement and super-resolution algorithms of thermal images, focused only on sensors onboard satellite, there are other options different from pansharpening algorithms. Processes called downscaling land surface temperature (DLST) try to obtain high-resolution thermal images from satellite data [11,12].

Apart from hardware solutions, we consider pansharpening algorithms applied to thermal imaging to be the best method to improve image resolution where simultaneous visible spectrum imaging is available.

New pansharpening algorithms known as hyperpansharpening are currently available for fusing several high-resolution images with multi and hyperspectral images [13,14,15,16,17]. These new algorithms are not studied in this analysis, as our aim is to relate our results with previous research on how to improve the resolution of thermal images with pansharpening algorithms [4,5,18,19].

The main aim of our study is to analyse the quality of the various pansharpening methods when using thermal images, based on the composition of a pseudo-multispectral (PS-MS) image from the raw thermal image. When fused with other much higher-resolution images using pansharpening methods, these PS-MS images will provide enhanced thermal imaging with a higher resolution than the original thermal image. This is the first quantitative analysis of UAV thermal images until now, and it allows a far more objective criterion for the algorithm for selecting the method to be used when processing this type of images.

In our work we have studied over ten pansharpening algorithms used in satellite image pansharpening from the two main families in order to determine their possibilities, performance, and results when used in thermal imaging. We apply our study to the case of UAVs, where the resolution and close geometry of these devices substantially modifies the results, and where it is necessary to fuse images from a range of image sensors. This research confirms the performance of pansharpening algorithms, and analyses the final products by means of numerical quality imaging indices to establish their quality. Prior research on thermal image pansharpening did not monitor performance in measurable and comparable numerical parameters, and as the findings were based merely on visual observation, it was impossible to ensure the quality in further processes and analyses using these enhanced images.

The rest of this manuscript is organized as follows. Section 2 introduces the pansharpening algorithms tested, the sample data and the testing methodology, which are the basis of the proposed qualitative assessment method. Finally, the algorithms are evaluated. Section 3 presents the quantitative quality results obtained for the selected algorithms. Section 4 contains a discussion of these results and their implications. The work is concluded in Section 5.

## 2. Materials and Methods

Multispectral images are composed of spectral bands that represent different parts of the electromagnetic spectrum. The typical bands in these images correspond to “colours” from the visible spectrum: red, green, and blue. Other common bands in multispectral imaging denote separate parts of the infrared spectrum such as near-infrared (NIR) or short-wavelength infrared (SWIR). The part known as long wave infrared (LWIR) in the infrared spectrum corresponds to thermal imaging. Other bands commonly found in multispectral imaging are from the ultraviolet spectrum.

In summary, we can define a multispectral image as the compound of multiple images (usually between 3 and 15) corresponding to different parts of the spectrum or “colours”.

Thermal images are usually processed using various masks or colour charts to form a false colour image. This aids the visual analysis and makes it easier for users to interpret. The colour chart most commonly used in these images shows lower temperatures in cold colours such as blue and violet, and higher temperatures in colours like yellow, orange and red. Although this is merely an artificial representation of the value of the raw grayscale image, it helps us form our pseudo-multispectral image (*PS-MS*).

**Figure 1:**
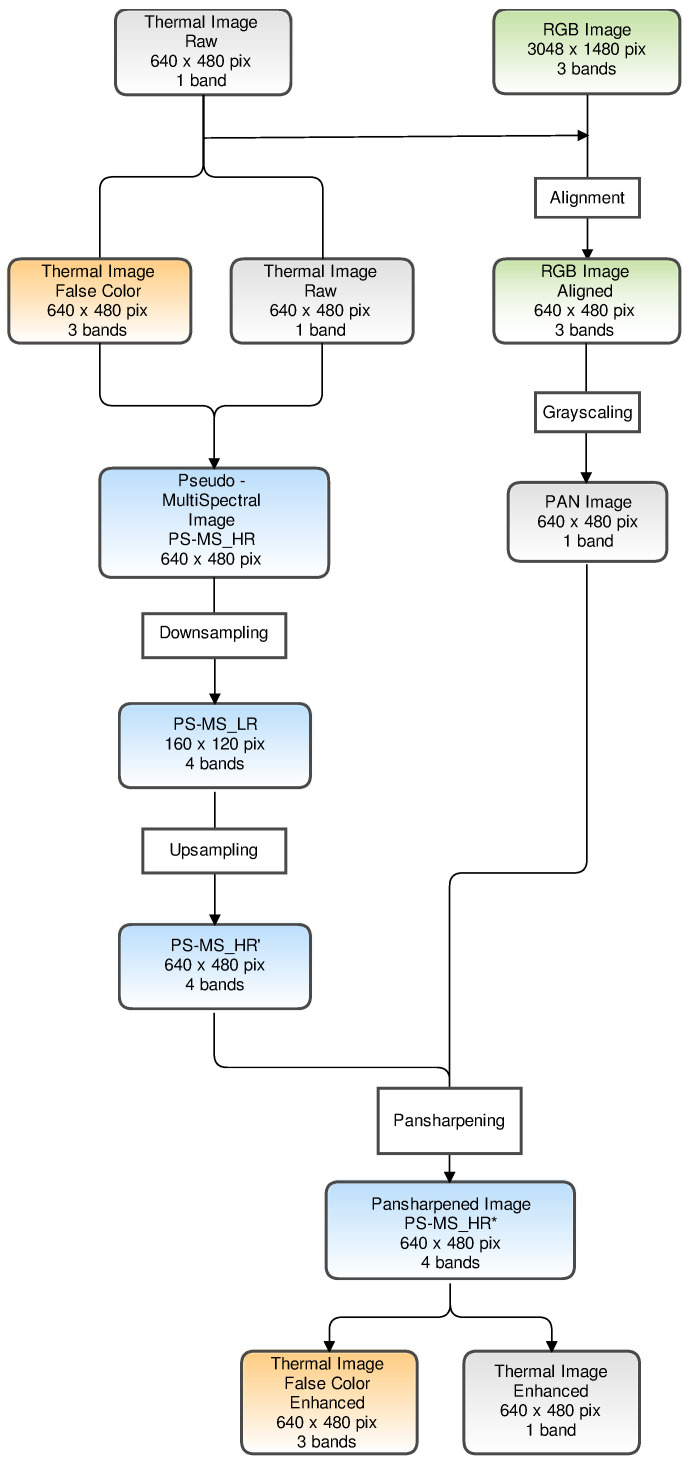
Proposed workflow for the pansharpening assessment methodology of thermal and RGB images with pseudo-multispectral image composition, and the down- and upsampling resolution steps.

Our PS-MS image is composed of four bands: three bands (red, green and blue) from the false colour image and the band corresponding to the original thermal image in grayscale. To clarify our assessment methodology, Figure 1 shows the workflow we followed, from the raw thermal image to the pansharpened final products.

To verify the performance of the various pansharpening algorithms we started by obtaining the PS-MS image in low resolution (PS-MS_LR), as the image was taken with a lower resolution sensor (160 × 120 pixels). This is done by applying a gaussian pyramidal algorithm, with ratio = 4 and σ = 4/3 (downsampling) [20].

The visible spectrum RGB images must have approximately the same field of view as the raw thermal image. The alignment step consists of calculating an affine transformation, identifying common points from both images, and then applying it. Most popular image alignment algorithms are feature-based and include keypoints detectors and local invariant descriptors [21]. In this work, we have implemented an ORB alignment algorithm [22,23], calculating the parameters which define the affine transformation.

The thermal and visible spectrum images are now coherent. The next step is to express the three RGB image bands in a single band in grayscale (grayscaling step). This is the panchromatic image (*PAN*) that is required for every pansharpening algorithm [16]. This PAN image is a simulation of the image that would be taken with a single specific sensor with a spectral range from blue to red (400–700 nm). As we are not using a high-resolution multispectral image, we do not analyse the hyperpansharpening algorithms.

The PAN image in our work has a resolution of 640 × 480 pixels (the original was 3048 × 1480 pixels). This will help us in later steps, as our aim is to analyse the pansharpening of the simulated low-resolution pseudo-multispectral image (160 × 120 pixels) and compare the final product with the original pseudo-multispectral image, with a resolution of 640 × 480 pixels.

The prior step for all the pansharpening algorithms analysed is the conversion of the low-resolution images to match the resolution of the panchromatic image. The size of both the low-resolution pseudo-multispectral (PS-MS_LR) and panchromatic image must match. This is achieved by applying a nearest neighbour-upsampling method, which yields a PS-MS_HR’ image (upsampling).

We can now apply all the selected pansharpening algorithms to obtain the enhanced resolution image PS-MS_HR*, formed by four bands: three RGB false colour bands and one thermal band (Figure 1). For further analysis, we split the final pansharpened image PS-MS_HR* into two images: one false colour and one thermal image.

### 2.1. Pansharpening Algorithms

Pansharpening algorithms belong to the image fusion branch of computer imaging, and their purpose is to enhance low-resolution images using images from another sensor with a higher resolution. It should be noted that both images must show the same object and have the same field of view. Two well-defined families of pansharpening algorithms are described in the scientific literature, mainly differentiated by whether their approach to the problem is spatial or spectral.

Algorithms known as COMPONENT SUBSTITUTION (CS) are based on the low resolution (LR) image colour space transformation in another space, and disassociate spatial and spectral information. The spatial information is then substituted by the information from the high resolution (HR) image. The process ends with the inverse colour space transformation. CS algorithms are global, as they act uniformly throughout the entire extension of the image [24].

MULTIRESOLUTION ANALYSIS (MRA) methods use linear space-invariant digital filtering of the HR image to extract the spatial details to be added to the LR bands [25].

MRA-based techniques substantially split the spatial information from the LR bands and the HR image into a series of bandpass spatial frequency channels. The high-frequency channels are inserted into the corresponding channels of the interpolated LR bands [25].

Our work focuses on the following algorithms from among all the pansharpening methods:

- IHS: Fast Intensity-Hue-Saturation (FIHS) image fusion [26].
- PCA: Principal Component Analysis [3].
- BDSD: Band-Dependent Spatial-Detail with local parameter estimation [27].
- GS: Gram Schmidt (Mode 1) [28].
- PRACS: Partial Replacement Adaptive Component Substitution [29].
- HPF: High-Pass Filtering with 5 × 5 box filter for 1:4 fusion [3]
- SFIM: Smoothing Filter-based Intensity Modulation (SFIM) [30,31].
- INDUSION: Decimated Wavelet Transform (DWT) using an additive injection model [32].
- MTF-GLP: Generalized Laplacian Pyramid (GLP) [33] with Modulation Transfer Function (MTF) matched filter [34] with unitary injection model.
- MTF-GLP-HPM: GLP with MTF-matched filter [34] and multiplicative injection model [35].
- MTF-GLP-HPM-PP: GLP with MTF-matched filter [34], multiplicative injection model and post-processing [36].
- MTF-GLP-ECB: MTF-GLP with Enhanced Context-Based model (ECB) algorithm [34].

Algorithms IHS, PCA, GS, BDSD, and PRACS belong to the CS category, and we selected HPF, SFIM, INDUSION and the different MTF variations from the group of MRA algorithms. All these algorithms have been computed using a MATLAB library distributed by Vivone et al. [37].

After establishing the scope of our study, we then define the characteristics to be met by the final products to ensure an adequate quantitative assessment. These properties are defined by Wald’s protocol [38].

### 2.2. Wald’s Protocol

Before proceeding, the images resulting from the pansharpening methods must be evaluated in terms of quantitative quality indices, as a visual inspection of the result is insufficient to determine their suitability.

The research community accepts Wald’s protocol [38,39] as establishing the essential properties of the products of image fusion algorithms where possible. These are as follows, as expressed by Aiazzi et al. [25]

**Theorem** **1.** *Consistency: any fused image Â, once degraded to its original resolution, should be as identical as possible to the original image A*

**Theorem** **2.** *Synthesis: any image Â fused by means of a high-resolution (HR) image should be as identical as possible to the ideal image $A_{I}$ that the corresponding sensor, if it exists, would observe at the resolution of the HR image.*

**Theorem** **3.** *The multispectral vector of images Â fused by means of a high-resolution (HR) image should be as identical as possible to the multispectral vector of the ideal images $A_{I}$ that the corresponding sensor, if it exists, would observe at the spatial resolution of the HR image.*

As the original image $A_{I}$ is available in our research, we can comply with Theorems 2 and 3 of Wald’s protocol.

The quality of the final products of fusion imaging must then be assured. Visual checking may be necessary, but an objective numerical comparison is compulsory. Various image fusion quality indices have been proposed to assess the quality of the fusion image procedures.

### 2.3. Quality Metrics

Fusion imaging quality indices aim to measure spatial and spectral distortion based on different statistical expressions with variations between them. They examine one particular aspect: some focus on the quality of the spatial reconstruction, whereas others are designed to evaluate the spectral variation.

Some terms must be defined in order to explain the indices involved, some terms must be defined. Let us define High Resolution Pseudo-Multiespectral image PS-MS_HR as $X\in R^{B\times P}$, with *B* bands and *P* pixels. $X={\left[x_{1},\ldots ,x_{B}\right]}^{T}=\left[x_{1},\ldots ,x_{P}\right]$, where $x_{i}\in R^{P\times 1}$ is the *i*th band (i=1,…,B) and $x_{j}\in R^{B\times 1}$ is the feature vector of the *j*th pixel (j=1,…,P). $X^{*}$ is the resulting image product after the pansharpening method (PS-MS_HR*). All the indices have been computed using the SEWAR python package [40].

#### 2.3.1. Root Mean Squared Error (RMSE)

The computed root mean squared error of the two images reveals the variation in the pansharpening process [41]. RMSE expresses both the spectral and spatial distortion of the improved image. The optimal value of RMSE is zero.

(1) $$\mathrm{RMSE}\left(X,X^{*}\right)=\sqrt{\frac{1}{B}\sum_{i=1}^{B}{∥x_{i}-x_{i}^{*}∥}^{2}}$$

RMSE may lead to an error in interpretation. It should be noted that under human perception, images that are unquestionably different may have an identical RMSE. Although the RMSE statistic may not be the most specific for expressing quality results, it contributes to the global vision with more complex indices such as SAM, ERGAS, etc. [42].

#### 2.3.2. *Erreur Relative Globale Adimensionnelle de Synthèse* (ERGAS)

A more advanced image quality index than RMSE was proposed by Ranchin and Wald [39]. ERGAS is a global statistic expressing the quality of the enhanced resolution image. ERGAS measures the transition between spatial and spectral information [43].

(2) $$\mathrm{ERGAS}\left(X,X^{*}\right)=100d\sqrt{\frac{1}{B}\sum_{i=1}^{B}\frac{{∥x_{i}-x_{i}^{*}∥}_{2}^{2}}{{\left(\frac{1}{P}{1_{P}}^{T}x_{i}\right)}^{2}}}$$

where *d* is the resolution ratio between the LR image and HR image (d=4, in this case), and $1_{P}={\left[1,\ldots ,1\right]}^{T}\in R^{P\times 1}$. ERGAS is the band-wise normalized root-mean-squared error multiplied by the GSD ratio in order to consider the difficulty of the fusion problem into consideration [44]. The optimal value of ERGAS is 0.

#### 2.3.3. Spectral Angle Mapper (SAM)

Another quality index, this time focused on spectral information, is the Spectral Angle Mapper SAM [44]. SAM measures the spectral distortion with the angle formed by two vectors of the spectrum of both images.

(3) $$\mathrm{SAM}\left(x_{j},{x^{*}}_{j}\right)=\arccos\left(\frac{x_{j}^{T}{x^{*}}_{j}}{{∥x_{j}∥}_{2}{∥{x^{*}}_{j}∥}_{2}}\right)$$

The equation determines the similarity between two spectra by calculating the angle between them and treating them as vectors in a space with a dimensionality equal to the number of bands [45]. The optimal value of SAM is zero. Here we express SAM as the average of all pixels in the image, in radians.

#### 2.3.4. Peak Signal to Noise Ratio (PSNR)

PSNR describes the spatial reconstruction in the final images [44], and is defined by the ratio between the maximum power of the signal and the power of the residual errors

(4) $$\mathrm{PSNR}\left(x_{i},x_{i}^{*}\right)=10\cdot \log_{10}\left(\frac{\max{\left(x_{i}\right)}^{2}}{{∥x_{i}-x_{i}^{*}∥}_{2}^{2}/P}\right)$$

where $\max(x_{i})$ is the maximum pixel value in *i*th band in the PS-MS_HR image.

A higher PSNR value implies a greater quality of the spatial reconstruction in the final image. If the images are identical, PSNR is equal to infinity.

#### 2.3.5. Universal Quality Index (UQI)

UQI estimates the distortion produced by combining three factors: correlation loss, luminance distortion and contrast distortion [46], as can be seen in the following equation.

(5) $$\mathrm{UQI}=\frac{\sigma_{xx^{*}}}{\sigma_{x}\sigma_{x^{*}}}\cdot \frac{2\bar{x}\bar{x^{*}}}{{\left(\bar{x}\right)}^{2}+{\left(\bar{x^{*}}\right)}^{2}}\cdot \frac{2\sigma_{x}\sigma_{x^{*}}}{\sigma_{x}^{2}+\sigma_{x^{*}}^{2}}$$

where $\sigma_{xx^{*}}=\frac{1}{P}\sum_{j=1}^{P}\left(x_{j}-\bar{x}\right)\left(x_{j}^{*}-\bar{x^{*}}\right)$, $\sigma_{x}=\sqrt{\frac{1}{P}\sum_{j=1}^{P}{\left(x_{j}-\bar{x}\right)}^{2}}$, $\sigma_{x^{*}}=$$\sqrt{\frac{1}{P}\sum_{j=1}^{P}{\left(x_{j}^{*}-\bar{x^{*}}\right)}^{2}}$, $\bar{x}=\frac{1}{P}\sum_{j=1}^{P}x_{j}$, and $\bar{x^{*}}=\frac{1}{P}\sum_{j=1}^{P}y_{j}$. 

UQI values move inside the [−1, 1] interval, where 1 is the optimal.

The quality indices have been computed separately for a more detailed analysis: false colour images and the image in grayscale corresponding to the fourth band in the PS-MS_HR and PS-MS_HR* images. This allows us to distinguish the transformation quality independently of the colour mask applied.

### 2.4. Datasets

Two different image datasets were built in order to test the performance of the pansharpening algorithms in thermal imaging. We started working with the FLIR ADAS dataset to evaluate the thermal quantification. This dataset is provided by FLIR thermal sensors brand and can be understood as a theoretical collection. For that reason, Illescas UAV was captured by us to evaluate and contrast the first dataset, this time focused on UAV specifically.

#### 2.4.1. FLIR ADAS Dataset

The FLIR Thermal Starter Dataset [47] was originally designed to supply a thermal image and a set of RGB images for training and validating neural networks for object detection. It provides thermal and RGB images simultaneously, making it optimal for applying pansharpening methods.

The dataset was acquired via a RGB and thermal camera mounted on a vehicle (car). It contains 14,452 annotated thermal images with 10,228 images sampled from short videos, and 4224 images from a continuous 144 s video. All videos were taken on streets and highways in Santa Barbara, CA, USA, under generally clear-sky conditions during both day and night.

Thermal images were acquired with a FLIR Tau2 (13 mm f/1.0, 45-degree horizontal field of view (HFOV) and a vertical field of view (VFOV) of 37 degrees). RGB images were acquired with a FLIR BlackFly at 1280 × 512 pixels (4–8 mm f/1.4–16 megapixel lens with the field of view (FOV) set to match Tau2). The cameras were 48 ± 2 mm apart in a single enclosure.

As both sensors were mounted on the same structure with different lenses and resolutions, a previous work of alignment is essential [48]. Image alignment (also known as image registration) is the technique of warping one image (or sometimes both images) to ensure the features in the two images line up perfectly so that both images show the same field. We calculated an affine transformation to resolve this by identifying clearly-distinguished common points in both images. The result of this transformation is that both images are now aligned in preparation for further pansharpening analysis.

Once the performance of the algorithms was confirmed, we obtained our own dataset with the requirements needed for our application, with a focus on aerial surveying.

#### 2.4.2. Illescas UAV Dataset

This second dataset comprised images taken from an unmanned aerial vehicle over an industrial building located in the town of Illescas (Toledo, Spain) on 13 August 2019 (40°8${}^{\prime }$41${}^{\prime \prime }$ N, 3°49${}^{\prime }$12${}^{\prime \prime }$ W).

The aerial vehicle was equipped with two sensors: a 4K RGB CMOS sensor with a resolution of 3840 × 2160 pixels; and an uncooled VOx microbolometer radiometric thermal infrared sensor with a pixel size of 17 micrometres. The thermal images have 640 × 512 pixels, spectral bands of between 7.5 and 13.5 micrometres, and a temperature sensitivity of 50 mK.

As with the FLIR ADAS dataset, an affine transformation must be computed to ensure both images are aligned before further analysis.

## 3. Results

Table 1, Table 2, Table 3 and Table 4 show a summary of the quality indices explained in Section 2.3 and calculated from the FLIR ADAS and Illescas UAV datasets. As stated above, these indices have been computed independently for false colour images and raw grayscale images to allow us to distinguish real performance without the influence of the false colour table. Bold values show the column best index value.

We have chosen a sample of 12 images from each dataset following our complete proposed workflow, and then computed all the quality indices with all the final products obtained from the sample 12 images from both datasets. The following values correspond to the mean values of the group and their dispersion expressed by their standard deviation.

**Figure A1:**
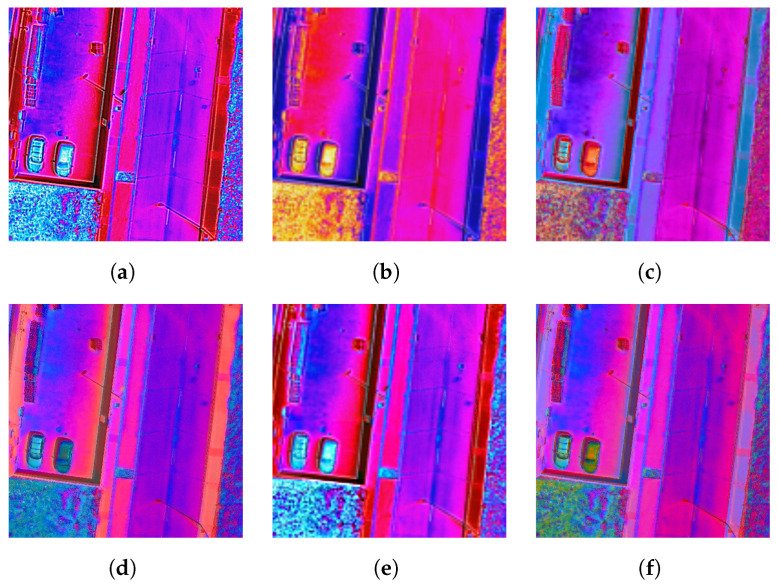

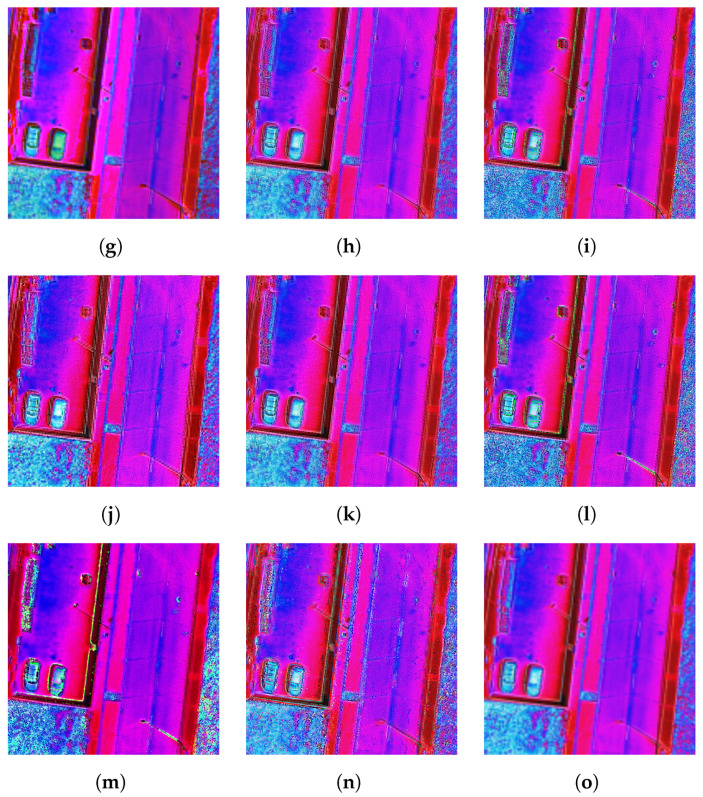
UAV Illescas dataset RGB: (**a**) Reference image; (**b**) Low-resolution upsampled image; (**c**) PCA; (**d**) IHS; (**e**) BDSD; (**f**) GS; (**g**) PRACS; (**h**) HPF; (**i**) SFIM; (**j**) INDUSION; (**k**) MTF-GLP; (**l**) MTF-GLP-HPM; (**m**) MTF-GLP-HPM-PP; (**n**) MTF-GLP-ECB; (**o**) MTF-GLP-CBD.

**Figure A2:**
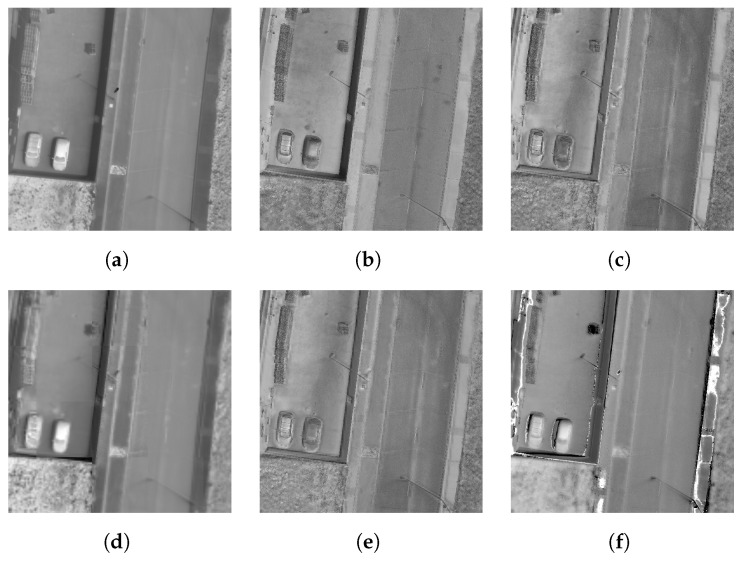

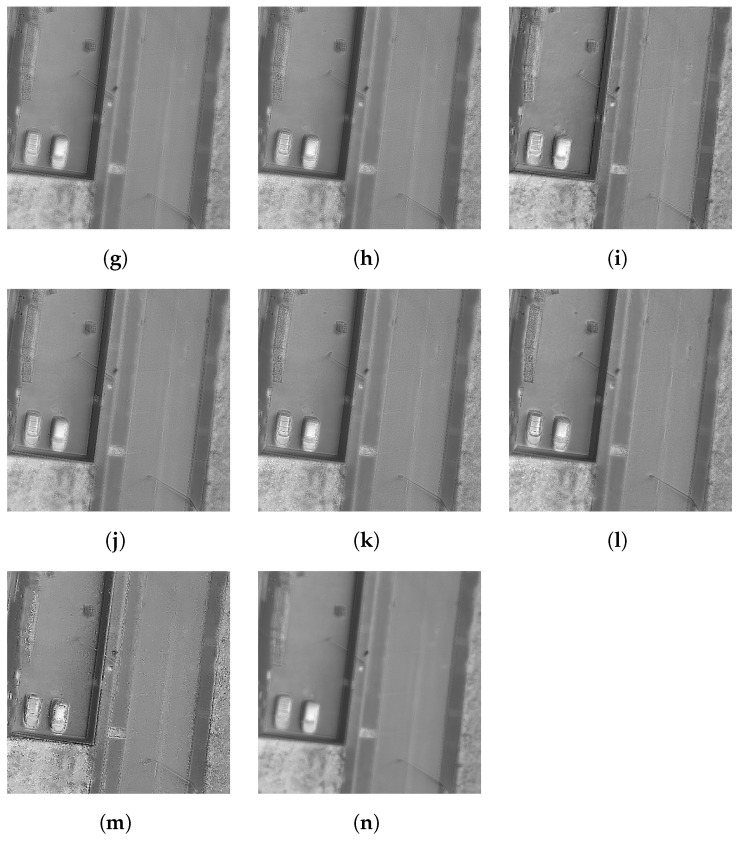
UAV Illescas dataset grayscale: (**a**) Reference image; (**b**). PCA, (**c**) IHS; (**d**) BDSD; (**e**) GS; (**f**) PRACS; (**g**) HPF; (**h**) SFIM; (**i**) INDUSION; (**j**) MTF-GLP; (**k**) MTF-GLP-HPM; (**l**) MTF-GLP-HPM-PP; (**m**) MTF-GLP-ECB; (**n**) MTF-GLP-CBD.

**Figure A3:**
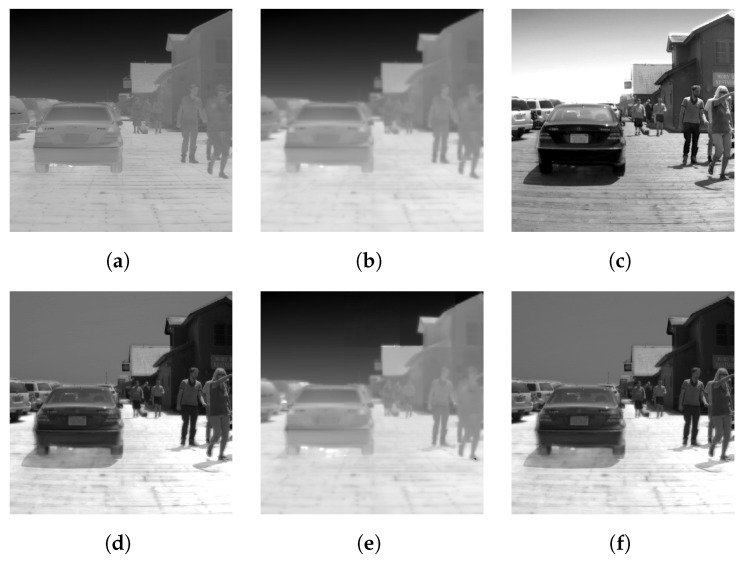

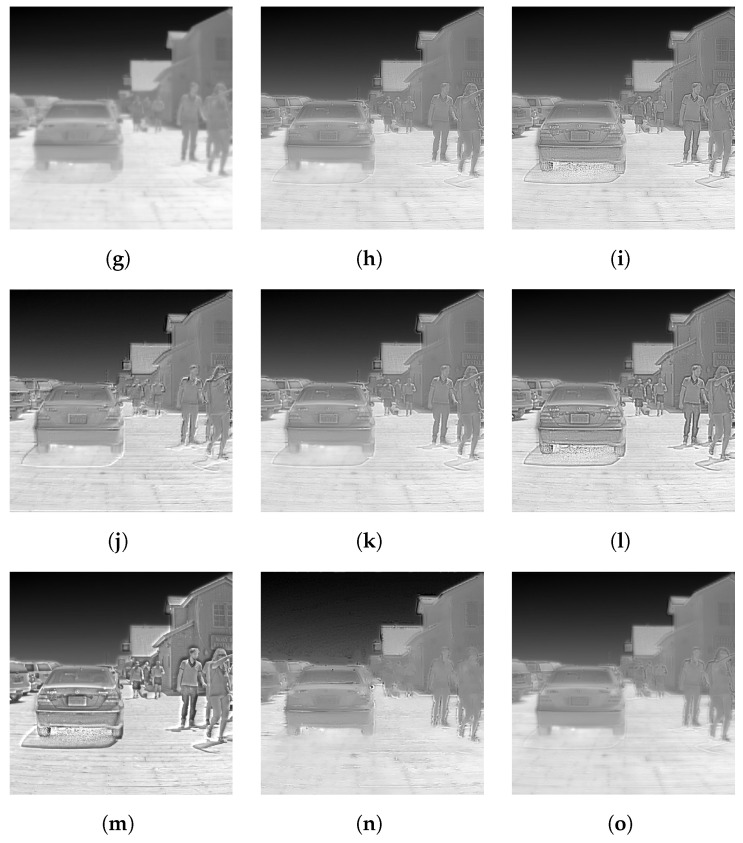
FLIR ADAS dataset [47] grayscale: (**a**) Reference image; (**b**) Low-resolution upsampled image; (**c**) PCA; (**d**) IHS; (**e**) BDSD; (**f**) GS; (**g**) PRACS; (**h**) HPF; (**i**) SFIM; (**j**) INDUSION; (**k**) MTF-GLP; (**l**) MTF-GLP-HPM; (**m**) MTF-GLP-HPM-PP; (**n**) MTF-GLP-ECB; (**o**) MTF-GLP-CBD.

**Figure A4:**
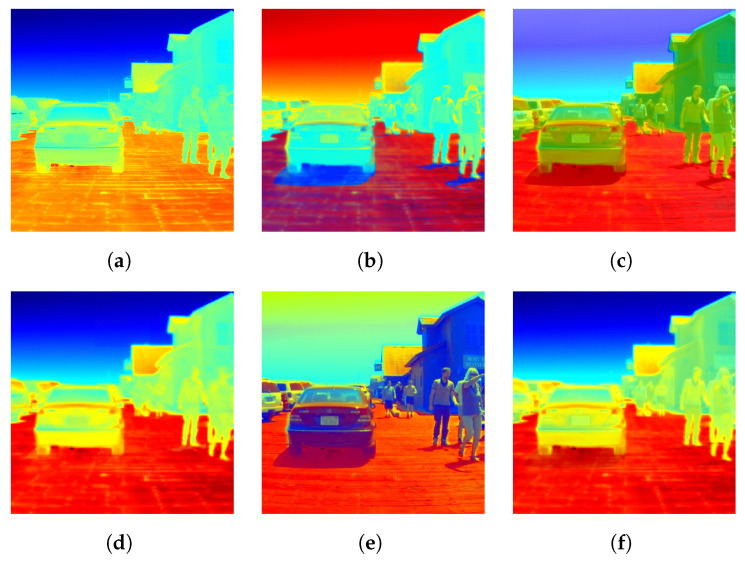

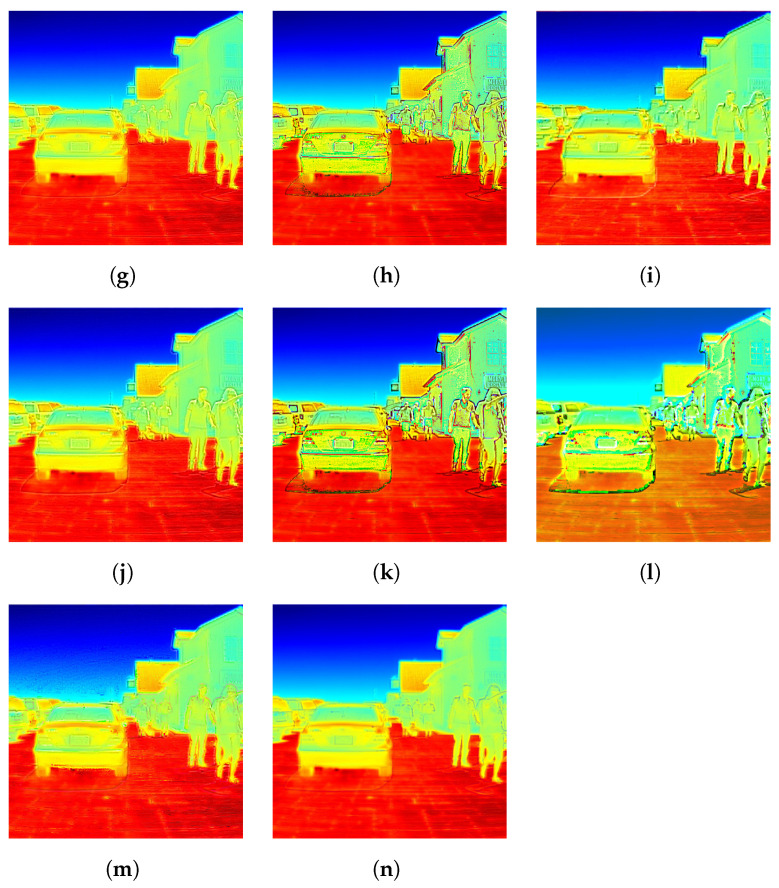
FLIR ADAS dataset [47] RGB: (**a**) Reference image; (**b**) PCA, (**c**) IHS; (**d**) BDSD; (**e**) GS; (**f**) PRACS; (**g**) HPF; (**h**) SFIM; (**i**) INDUSION; (**j**) MTF-GLP; (**k**) MTF-GLP-HPM; (**l**) MTF-GLP-HPM-PP; (**m**) MTF-GLP-ECB; (**n**) MTF-GLP-CBD.

Figure A1, Figure A2, Figure A3 and Figure A4 in the Appendix A show a composition of a sample image from each dataset: the original, the upsampled, and pansharpened images from every studied algorithm. We confirm that a visual analysis is insufficient to validate the final quality of the image fusion process.

**Figure 2:**
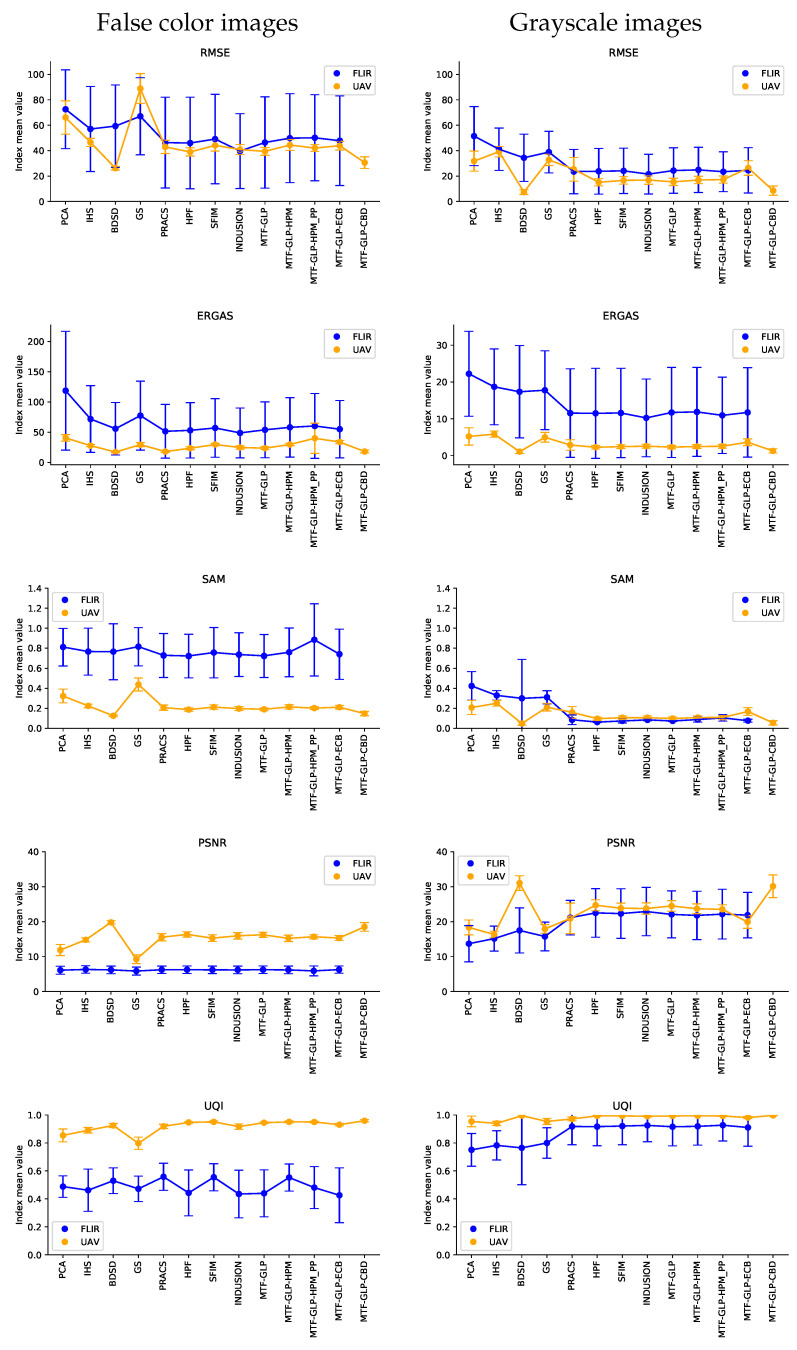
Graphic representation of computed quality indices. The different indices have been categorized in false color and grayscale. Dots represent quality index value, and vertical line length shows sample standard deviations. Graphics in ERGAS row are not directy comparable due to different y-axis scale.

Figure 2 contains a graphic representation of the values of the various indices to aid the interpretation of the results.

## 4. Discussion

The FLIR ADAS and Toledo UAV datasets were processed and analysed, with the following results:

- The results for the false colour and grayscale images are quantitatively different. Grayscale images perform better than false colour images, thus confirming our hypothesis of separating the image fusion products into false colour and grayscale. The values of the RMSE index obtained for the images in grayscale are similar or even lower than in researches in the same field (RMSE similar to 31) [9]. The final grayscale image should be chosen for the subsequent processes, even when the same or a different false colour table needs to be applied again
- Apart from certain specific values, the two different families of algorithms have a similar performance. Minor differences in the way the different algorithms process the data produce better results. One instance of this can be seen in the case of the CS family with the BDSD algorithm, which performs better than the rest of the family. Figure 2 also reveals homogeneity among the values in the MRA family in all the indices.
- In general, MRA algorithms perform better than CS methods in thermal imaging, except in the case of the Component Substitution BDSD algorithm in the Illescas UAV dataset (Table 4), which has the best values in almost all the quality indices (RMSE = 7.400, ERGAS = 1.084, SAM = 0.048, PSNR = 31.014, UQI = 0.995). Haselwimmer et al. [5] suggest the IHS algorithm to fuse thermal and RGB images. Our work confirms that IHS is not the best choice of algorithm. Among the CS methods, the BDSD algorithm achieves the best results.
- Radiometrically speaking, there is no single best choice. ERGAS and SAM indices appear similar in both cases, although the algorithms from the MRA family perform slightly better. This agrees with the general behaviour described for these algorithms in the literature [49]. The values obtained in the SAM index (SAM < 1) are even better than those from other works on multi- and hyperspectral data fusion (SAM > 1) [17].
- Spatial reconstruction is better in MRA methods. PSNR has higher values in both datasets, denoting a greater geometrical quality of the spatial details. Again, the BDSD algorithm is the best in terms of spatial reconstruction.
- Regarding the behavior of the datasets, the UAV dataset obtains better results in all indices, possibly due to the nature of the FLIR ADAS dataset. The lack of homogeneity between the distances to the objects, may explain the poorer performance of the pansharpening algorithms, and may also be the reason for higher dispersion values in the whole FLIR dataset. We could fix this by decomposing the images in subzones where the distances were homogeneous and analyzing their influence.
- Our work allows the use of thermal sensors with a lower resolution than other types of sensors used simultaneously in the same project, since this method enhances the resolution of the thermal images and homogenises their resolution. One limitation is that it depends on the resolution ratio between visible and thermal spectrum images. Here, a ratio of more than four may lead to unexpected artifacts and to the failure of processes [50].
- Although the results may vary depending on the false colour representation of the thermal information adopted, the validation by the grayscale band highlights the interest of further developments to adjust the parameters of the algorithms to adapt them specifically to infrared thermal images.

## 5. Conclusions

The use of certain pansharpening algorithms applied to thermal images has been tested in previous research. This work contains a complete review of a number of algorithms, and provides an in-depth study of thermal imaging pansharpening, with a numerical assessment.

We have validated the potential of pansharpening algorithms to enhance the resolution of thermal images with the help of higher-resolution visible spectrum RGB images. Algorithms from the two main pansharpening families have been tested on different datasets, and the quality of the results has been verified. This quantitative analysis allows us to make a critical comparison.

Our focus on UAV imaging suggests a primary application, as all UAV platforms have quite different sensor resolutions between the thermal and visible spectrum. This type of aerial platforms fitted with this type of sensors are already very useful in such key areas as volcanism, the detection of temperature changes as a possible parameter for forecasting future events, and the inspection of industrial electromechanical elements, where they can be a key factor in preventing system malfunctions. The availability of a more accurate estimate of the quality of thermal image pansharpening algorithms will make it easier to develop more reliable automatic remote sensing systems.

## Figures and Tables

**Table 1:** Quality indices for the False Colour Thermal Pansharpened images of the FLIR ADAS dataset for each pansharpening algorithm tested.

Algorithm	RMSE		ERGAS		SAM		PSNR		UQI	
	Mean	Std	Mean	Std	Mean	Std	Mean	Std	Mean	Std
PCA	72.565	31.019	118.645	98.245	0.811	0.187	6.117	1.147	0.488	0.077
IHS	57.025	33.458	71.754	55.219	0.766	0.234	**6.318**	1.080	0.462	0.151
BDSD	59.318	32.405	55.732	43.369	0.765	0.279	6.205	1.095	0.530	0.092
GS	67.068	30.342	77.425	57.028	0.815	0.190	5.866	1.151	0.472	0.091
PRACS	46.300	35.710	51.463	44.451	0.728	0.219	6.233	1.067	0.558	0.097
HPF	46.014	36.066	53.070	45.810	**0.722**	0.217	6.254	1.081	0.443	0.164
SFIM	49.102	35.220	56.999	48.461	0.756	0.251	6.208	1.078	**0.555**	0.097
INDUSION	**39.666**	29.428	**48.817**	41.172	0.736	0.218	6.196	1.078	0.435	0.170
MTF-GLP	46.426	35.898	53.918	46.157	0.723	0.214	6.255	1.072	0.440	0.168
MTF-GLP-HPM	49.816	35.031	58.035	49.192	0.759	0.243	6.186	1.080	0.553	0.097
MTF-GLP-HPM_PP	50.127	33.860	60.282	53.623	0.884	0.360	5.913	1.400	0.481	0.150
MTF-GLP-ECB	47.818	35.316	54.997	47.433	0.740	0.250	6.277	1.035	0.426	0.196

**Table 2:** Quality indices for the False Colour Thermal Pansharpened images of the Illescas UAV dataset for each pansharpening algorithm tested.

Algorithm	RMSE		ERGAS		SAM		PSNR		UQI	
	Mean	Std	Mean	Std	Mean	Std	Mean	Std	Mean	Std
PCA	66.084	13.109	40.524	5.339	0.323	0.069	11.883	1.597	0.854	0.046
IHS	46.524	3.162	27.716	1.736	0.224	0.018	14.798	0.599	0.891	0.020
BDSD	**26.167**	1.415	**17.235**	1.079	**0.125**	0.008	**19.789**	0.472	0.925	0.014
GS	88.872	11.741	29.372	3.428	0.438	0.065	9.241	1.253	0.798	0.044
PRACS	42.932	5.172	17.977	1.444	0.207	0.026	15.537	1.026	0.919	0.015
HPF	38.962	3.236	23.463	2.332	0.187	0.016	16.350	0.758	0.947	0.006
SFIM	44.139	4.425	29.593	1.927	0.212	0.023	15.284	0.961	0.951	0.007
INDUSION	40.867	4.060	24.698	2.753	0.197	0.020	15.951	0.932	0.917	0.020
MTF-GLP	39.435	3.128	23.585	2.266	0.190	0.015	16.243	0.726	0.945	0.006
MTF-GLP-HPM	44.432	4.259	29.617	1.790	0.214	0.022	15.222	0.913	0.951	0.007
MTF-GLP-HPM_PP	42.047	2.671	39.971	25.048	0.202	0.014	15.675	0.584	0.950	0.006
MTF-GLP-ECB	43.876	3.439	33.677	2.671	0.211	0.018	15.314	0.699	0.931	0.009
MTF-GLP-CBD	30.612	4.561	18.023	2.340	0.147	0.021	18.503	1.231	**0.959**	0.008

**Table 3:** Quality indices for the Grayscale Thermal Pansharpened images of the FLIR ADAS dataset for each pansharpening algorithm tested.

Algorithm	RMSE		ERGAS		SAM		PSNR		UQI	
	Mean	Std	Mean	Std	Mean	Std	Mean	Std	Mean	Std
PCA	51.432	23.291	22.231	11.541	0.424	0.143	13.692	5.176	0.751	0.117
IHS	41.121	16.668	18.692	10.303	0.329	0.048	15.152	3.569	0.783	0.105
BDSD	34.386	18.594	17.346	12.541	0.300	0.390	17.486	6.454	0.765	0.264
GS	38.844	16.334	17.767	10.694	0.311	0.065	15.756	4.101	0.800	0.109
PRACS	23.521	17.460	11.548	12.030	0.085	0.048	21.132	4.960	0.918	0.131
HPF	23.769	17.956	11.491	12.244	**0.062**	0.004	**22.499**	6.956	0.917	0.137
SFIM	24.098	17.842	11.582	12.136	0.073	0.020	22.307	7.105	0.921	0.134
INDUSION	**21.525**	15.616	**10.243**	10.554	0.084	0.008	22.892	6.929	0.926	0.117
MTF-GLP	24.326	17.910	11.718	12.228	0.073	0.005	22.074	6.728	0.916	0.137
MTF-GLP-HPM	24.854	17.780	11.879	12.088	0.087	0.025	21.780	6.907	0.919	0.134
MTF-GLP-HPM_PP	23.432	15.712	10.940	10.374	0.104	0.032	22.146	7.108	**0.927**	0.113
MTF-GLP-ECB	24.489	17.868	11.733	12.143	0.076	0.014	21.870	6.508	0.911	0.134

**Table 4:** Quality indices for the Grayscale Thermal Pansharpened images of the Illescas UAV dataset for each pansharpening algorithm tested.

Algorithm	RMSE		ERGAS		SAM		PSNR		UQI	
	Mean	Std	Mean	Std	Mean	Std	Mean	Std	Mean	Std
PCA	31.774	7.927	5.209	2.353	0.208	0.071	18.353	2.127	0.954	0.038
IHS	39.167	4.122	5.837	0.851	0.250	0.028	16.322	0.939	0.940	0.015
BDSD	**7.400**	1.933	**1.084**	0.504	**0.048**	0.017	**31.014**	2.107	0.995	0.007
GS	32.743	4.492	4.974	1.326	0.211	0.038	17.915	1.242	0.954	0.022
PRACS	25.352	9.352	2.885	1.413	0.159	0.058	20.934	4.354	0.972	0.014
HPF	15.108	2.883	2.274	0.468	0.096	0.016	24.693	1.569	0.994	0.003
SFIM	16.610	2.933	2.436	0.504	0.106	0.017	23.847	1.436	0.994	0.002
INDUSION	16.848	3.282	2.565	0.521	0.106	0.018	23.754	1.609	0.991	0.004
MTF-GLP	15.441	2.866	2.315	0.474	0.098	0.016	24.496	1.525	0.993	0.003
MTF-GLP-HPM	16.892	2.885	2.471	0.509	0.107	0.017	23.693	1.388	0.994	0.002
MTF-GLP-HPM_PP	17.210	2.770	2.540	0.517	0.109	0.017	23.519	1.322	0.993	0.003
MTF-GLP-ECB	26.334	5.769	3.640	0.922	0.168	0.039	19.917	1.826	0.982	0.008
MTF-GLP-CBD	8.567	3.627	1.283	0.573	0.054	0.021	30.125	3.244	**0.997**	0.002

## Data Availability

Not applicable.
